# Obsessive-Compulsive Disorder and the Postpartum Period: A Clinical Analysis

**DOI:** 10.1192/j.eurpsy.2025.1720

**Published:** 2025-08-26

**Authors:** A. Rodríguez-Quiroga

**Affiliations:** Psychiatry, Hospital Universitario Infanta Leonor, Madrid, Spain

## Abstract

**Introduction:**

The postpartum period is a crucial stage in women’s lives, characterized by significant physical, emotional, and psychological changes. Among the mental complications that can arise during this stage, postpartum Obsessive-Compulsive Disorder (OCD) has gained increasing attention due to its significant impact on the quality of life of mothers and their ability to care for their newborns.

**Objectives:**

This study aims to analyze the prevalence, etiology, clinical manifestations, and therapeutic approaches of OCD in the postpartum period.

**Methods:**

The study reviews existing literature on the prevalence of postpartum OCD, which varies between 2% and 16.9%. It examines symptoms such as intrusive thoughts of harm towards the baby, repetitive checking behaviors, and cleaning compulsions. The study also identifies risk factors including personal or family history of OCD, traumatic experiences, and high-stress levels, as well as the influence of hormonal fluctuations and neurobiological changes on the vulnerability to postpartum OCD. The therapeutic approaches reviewed include pharmacological interventions, primarily with selective serotonin reuptake inhibitors (SSRIs), and psychological therapies, including cognitive-behavioral therapy (CBT) specialized in OCD.

**Results:**

The combination of pharmacological and psychological strategies shows promise in reducing symptoms and improving the overall functioning of patients. Early detection and adequate treatment of postpartum OCD are essential to prevent long-term complications, promoting the well-being of mothers and their families. The study underscores the need for further research and awareness about postpartum OCD to develop more effective mental health policies and specific support programs.

**Conclusions:**

This study underscores the need for further research and increased awareness about postpartum OCD. Developing more effective mental health policies and specific support programs is crucial to address this condition. Enhanced understanding and intervention can significantly improve outcomes for affected mothers and their families.

**Disclosure of Interest:**

A. Rodríguez-Quiroga Grant / Research support from: The conference registration will be funded by Adamed.

